# Asynchrony of ovule primordia initiation in *Arabidopsis*

**DOI:** 10.1242/dev.196618

**Published:** 2020-12-23

**Authors:** Shi-Xia Yu, Lv-Wen Zhou, Li-Qin Hu, Yu-Tong Jiang, Yan-Jie Zhang, Shi-Liang Feng, Yuling Jiao, Lin Xu, Wen-Hui Lin

**Affiliations:** 1 Joint International Research Laboratory of Metabolic & Developmental Sciences, School of Life Sciences & Biotechnology, Joint Center for Single Cell Biology, Shanghai Jiao Tong University, Shanghai 200240, China; 2 Faculty of Mechanical Engineering and Mechanics, Ningbo University, Ningbo, Zhejiang 315211, China; 3 State Key Laboratory of Plant Genomics, Institute of Genetics and Developmental Biology, Chinese Academy of Sciences, and National Center for Plant Gene Research, Beijing 100101, China; 4 College of Life Sciences, University of Chinese Academy of Sciences, Beijing 100049, China; 5 National Key Laboratory of Plant Molecular Genetics, CAS Center for Excellence in Molecular Plant Sciences, Institute of Plant Physiology and Ecology, Chinese Academy of Sciences, Shanghai 200032, China

**Keywords:** Ovule initiation, Asynchrony, Auxin, Brassinosteroid

## Abstract

Plant ovule initiation determines the maximum of ovule number and has a great impact on the seed number per fruit. The detailed processes of ovule initiation have not been accurately described, although two connected processes, gynoecium and ovule development, have been investigated. Here, we report that ovules initiate asynchronously. The first group of ovule primordia grows out, the placenta elongates, the boundaries of existing ovules enlarge and a new group of primordia initiates from the boundaries. The expression pattern of different marker genes during ovule development illustrates that this asynchronicity continues throughout whole ovule development. PIN-FORMED1 polar distribution and auxin response maxima correlate with ovule primordia asynchronous initiation. We have established computational modeling to show how auxin dynamics influence ovule primordia initiation. Brassinosteroid signaling positively regulates ovule number by promoting placentae size and ovule primordia initiation through strengthening auxin response. Transcriptomic analysis demonstrates numerous known regulators of ovule development and hormone signaling, and many new genes are identified that are involved in ovule development. Taken together, our results illustrate that the ovule primordia initiate asynchronously and the hormone signals are involved in the asynchrony.

## Abstract (precis)

**Summary:** Ovule primordia initiation, which determines the maximum ovule number and subsequent seed number in *Arabidopsis*, is asynchronous and is regulated by PIN1 polar distribution and the auxin response.

## INTRODUCTION

The formation of lateral organ primordia is a significant event in the growth and development of plants and animals. Plants initiate lateral organ primordia continuously and at regular positions from the growing tip; these processes are strictly regulated by plant hormones and other key regulators. Plant lateral organ primordia include the primordia of lateral root, leaf, and flower (Steeves and Sussex, 1989; Benková et al., 2003; Heisler et al., 2005), and ovule primordia initiation could be considered as another lateral organ initiation event (Cucinotta et al., 2014). It determines the maximum ovule number and also directly affects seed number per fruit and seed yield, as the ovule is the precursor of seed. In *Arabidopsis*, ovule primordia protrude from placentae: the tissue that develops from the carpel margin meristem in the gynoecium during flower development. Flower organ development is regulated by ABCDE genes, which specify four whorls of flower organs and ovules (Goto et al., 2001; Theißen, 2001; Pinyopich et al., 2003). The innermost whorl, i.e. the female reproductive organs, consists of two fused carpels that develop into four placentae (Bowman et al., 1991).

Previous studies have divided floral developmental process into 20 stages; ovule development mainly occurs during stages 9-12 (Smyth et al., 1990; Bowman et al., 1991; Robinson-Beers et al., 1992; Cucinotta et al., 2014). Ovules initiate in stage 9, megaspore mother cells (MMCs) identify at stage 10, megasporogenesis and integument initiation happen at stage 11, and the embryo sac develops at stage 12 (Bowman et al., 1991; Robinson-Beers et al., 1992). In addition, ovule development is further divided into four steps in more detail: early ovule development, megasporogenesis, megagametogenesis and post-fertilization development. Ovules protrude at ovule developmental stage 1-I, as described for the corresponding flower developmental stage 8 (Schneitz et al., 1995). However, many studies have reported that the ovule protrudes mainly at floral stage 9 (Bowman et al., 1991; Robinson-Beers et al., 1992; Cucinotta et al., 2014). So far, there are relatively few descriptions of early ovule development, including ovule identity and initiation, due to the intractable materials, limited technologies and narrow developmental phase.

Some genes have been reported to regulate ovule identity, and to influence ovule initiation and development, and thus ovule number and seed number per silique. These genes include those encoding multiple MADS-box transcription factors and other regulators, such as AGAMOUS (AG), SEEDSTICK (STK), SHATTERPROOF1 (SHP1), SHP2 and SEPALLATA (SEP) (Bowman et al., 1989; Honma and Goto, 1996; Theißen et al., 1996; Favaro et al., 2003), and AINTEGUMENTA (ANT), BELL1 (BEL1), APETALA2 (AP2) and HUELLENLOS (HLL) (Modrusan et al., 1994; Reiser et al., 1995; Elliott et al., 1996; Schneitz et al., 1998; Brambilla et al., 2007). Multiple hormones are also involved in ovule development and regulate ovule number through influencing gynoecium development, carpel fusion and CMM formation, processes that affect placenta development and ovule identity (Colombo et al., 2008; Reyes-Olalde et al., 2013; Marsch-Martínez and de Folter, 2016; Zúñiga-Mayo et al., 2019), including auxin, brassinosteroid (BR), cytokinin (CK) and gibberellin (GA) (Cucinotta et al., 2020). The weak mutant of the auxin efflux facilitator PIN-FORMED1 (PIN1), *pin1-5*, displays a reduction in ovule number (Bencivenga et al., 2012). BR-deficient or -insensitive mutants have decreased ovule number, and a BR signal enhanced mutant has enhanced ovule protraction and ovule number (Huang et al., 2013). The triple CK receptor mutant (*cre1-12 ahk2-2 ahk3-3*) has reduced ovule number, indicating that CK positively regulates ovule development and number (Higuchi et al., 2004; Bencivenga et al., 2012). The mutant of DELLA protein, a negative regulator of GA signaling, produces fruits with fewer seeds than those in wild type, indicating that loss of GA regulates ovule number (Gomez et al., 2018; Barro-Trastoy et al., 2020).

Auxin has been demonstrated to control primordia initiation in lateral root and shoot apical meristem (SAM) (Laskowski et al., 1995; Reinhardt et al., 2000). The combined action of differentially expressed and localized PIN1 proteins results in the formation of an auxin gradient, which mediates proper lateral root development (Casimiro et al., 2001; Benková et al., 2003). PIN1 polarity also causes changes in auxin levels to direct primordium development in the SAM (Heisler et al., 2005). In addition, the crosstalk between auxin and CK is essential for cell-type specification in the root, SAM and gynoecium. PIN1-GFP is expressed in the placenta and the epidermis cells of the ovule, and the DR5-GFP signal is visible after ovule protrusion (Galbiati et al., 2013). CUP-SHAPED COTYLEDON1 (CUC1) and CUC2 establish the boundaries of ovule primordia and control PIN1 expression in ovules (Galbiati et al., 2013; Gonçalves et al., 2015). Auxin triggers the expression of ANT and MONOPTEROS (MP), which are also required for ANT, CUC1 and CUC2 expression (Galbiati et al., 2013; Cucinotta et al., 2020). However, there is no detailed hypothesized model connecting PIN1 polarity, auxin maxima and whole ovule population initiation.

Overall, only a few studies focus on the earliest stage of ovule development. The detailed process of ovule primordia initiation remains unclear. Here, we report that ovules develop asynchronously in the same placenta, and ovule primordia initiate in different groups. Our results further show how PIN1 polarity and auxin gradient maxima in the placenta lead to the initiation of different groups of ovule primordia. We also establish a computational model describing the process of asynchronous ovule primordia initiation. BR signal promotes ovule number not only through increasing placenta length but also through stimulating ovule primordia initiation by strengthening auxin response. We also performed a microarray assay of the stage-specific gynoecium (removing the stigma and junction site of floral organs). Our comprehensive analysis revealed genes that functioned in ovule development. In conclusion, our results shed light on the detailed process of ovule primordia initiation and show how hormones integrate ovule primordia initiation and ovule development.

## RESULTS

### Ovule primordia initiate asynchronously on identical placentas

The protrusions, i.e. ovule primordia, are initiated by periclinal divisions of the subepidermal cells of the placenta at stage 9 (Bowman et al., 1991; Robinson-Beers et al., 1992). After a series of divisions, the ovule primordia differentiate and elongate along with the proximal-distal axis at stage 10 (Bowman et al., 1991; Robinson-Beers et al., 1992; Schneitz et al., 1995). Ovule development starts from stage 9, but the ovule shape differs between the beginning and end of stage 9, because stage 9 is relatively long. For easy observation and accurate description, we subdivided floral stage 9 into three substages: stages 9a, 9b and 9c, corresponding to the early, middle and late stage 9. If we consider the ovule primordium as a cylinder protruding from the placenta, there are at least three different shapes of ovule primordia at stage 9 based on the height and basal diameter of the cylinder. In the small/young ovule primordia (i.e. named O1 for convenience), the basal diameter of the ovule primordium is much larger than its height (Fig. S1A). In the middle age ovule primordia (O2), the basal diameter equals its height (Fig. S1B). In the large/old ovule primordia (O3), the basal diameter is much less than its height (Fig. S1C). O1, O2 and O3 ovule primordia are small-bump shaped, dome shaped and O3 finger shaped, respectively.

**Fig. 1.**
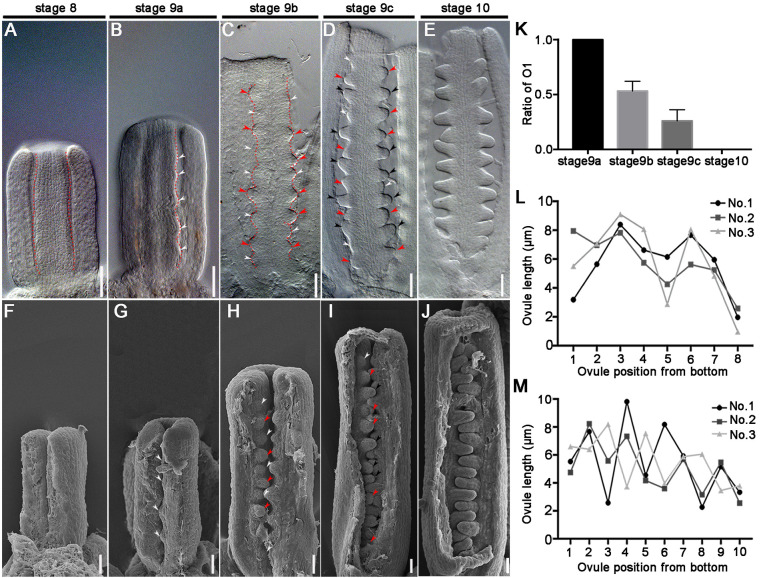
**Ovule primordia initiation process in *Arabidopsis* placentae.** (A-E) Differential interference contrast (DIC) images of *Arabidopsis* placentae at stages 8-10. Dotted lines highlight the placenta in A-C. (F-J) Scanning electron microscopy (SEM) images of *Arabidopsis* placentae at stages 8-10. (K) The ratio of young ovule primordia (O1) at stages 8a-10. The data are mean±s.d.; *n*>15 in every group (one-way ANOVA; *P*<0.0001). (L,M) The length of ovule protruding from the placenta at stage 9b (L) and stage 9c (M). Ovule orders are according to the position from the base: 1, 2 and 3 indicate different placentae. White arrowheads indicate the youngest batch of ovule primordia (O1) in B-D,G-I; red arrowheads indicate the older batch of ovule primordia (O2) in C,D,H,I; black arrowheads indicate the oldest batch of ovule primordia (O3) in D,I. Scale bars: 20 μm.

At stage 8, there are no protrusions on the placenta (Fig. 1A,F). At stage 9a, four to six ovule primordia initiate on each placenta, and all ovule primordia are small-bump shaped (O1) (Fig. 1B,G). At stage 9b, there are seven to nine ovule primordia on each placenta and old ovule primordia that initiated at stage 9a are dome shaped (O2), while the young ovule primordia are small-bump shaped (O1) (Fig. 1C,H). The number of ovule primordia peaks at ten to 14 at stage 9c, and the oldest batch of ovule primordia is finger shaped (O3), the younger batch is dome shaped (O2) and the youngest batch is small-bump shaped (O1) (Fig. 1D,I). At stage 10, all ovules are finger shaped (O3), and the number and shape of ovules at each placenta show only minor differences (Fig. 1E,J). The quantitative analysis demonstrated that ovule primordia were all in O1 condition at stage 9a, and the ratio of O1 reduced at stage 9b-9c, suggesting the ovule initiated at stage 9a, and grew to O2 or O3 when the new young ovule (O1 condition) protruded (Fig. 1K). Finally, all ovules grew to O2 and O3 conditions, and the young ovule primordia (O1) initiation stopped gradually, with O1 ovule absent by stage 10 (Fig. 1K). There are different ages and shapes of ovules in each placenta, which illustrates that ovule primordia initiation is asynchronous.

### Ovule primordia initiate in different groups

For determining the initiation order of different ovule primordia, we observed the protrusion process in many placentae. Photographic analyses showed that the larger and smaller (O2 and O1 or O3 and O2) ovules arranged alternately in the placenta (Fig. 1C,D,H,I,L,M; Fig. S1D-F). But there was not always one smaller ovule between every two larger ovules because there was sometimes insufficient space for new ovule primordia between each two larger ovules (Fig. 1L,M; Fig. S1D-F). Generally, each ovule primordium has a different size and shape from that of its neighbors.

Although the ovule primordia initiate from the placenta in groups, ovule primordia belonging to the same group do not appear identical in size and shape (Fig. 1B,G). We decided to observe the largest ovule primordium out of six ovule primordia in every placenta at stage 9a, as this must be the ovule that initiates first in the placenta (assuming all ovules grow at a similar speed) (Fig. S1D). Observing this first ovule primordium is difficult because it initiates over a very short period of time. For easy statistics, we divided the placenta into the upper, middle and lower parts. We found that 45.5% of first protrusions appeared in the lower part, about 47.6% in the middle part and about 6.9% in the top part (Fig. S1G). These results suggest that the first ovule primordia initiate mainly from the middle and lower parts of the placenta.

### CUC3, PIN1 and R2D2 signals indicate asynchronous initiation of ovule primordia

**Fig. 2.**
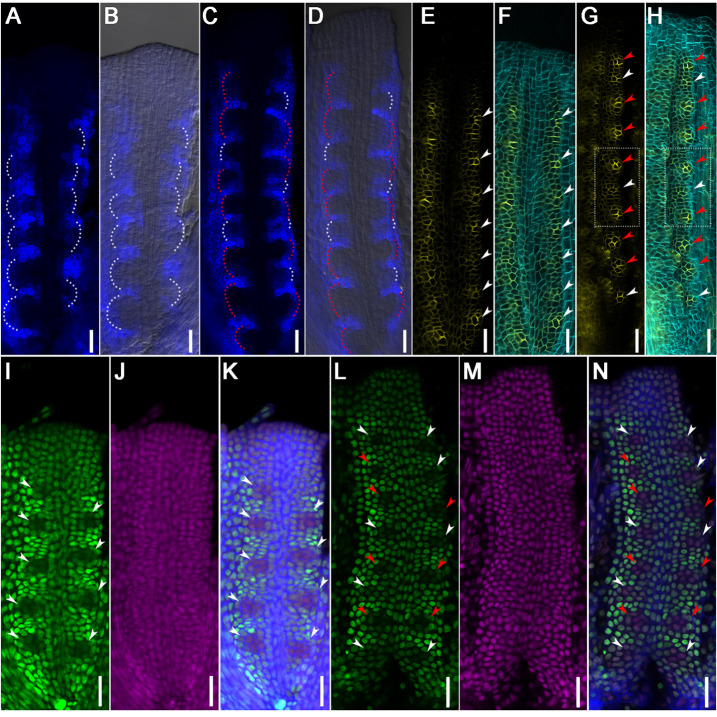
**The signals of different markers in the process of ovule primordia initiation.** (A-D) Distribution of *ProCUC3::CFP* in placentae at floral stage 9a (A,B) and stage 9b (C,D). (B,D) Images merged with the bright-field images. White dotted lines highlight the young batch of ovule primordia in A-D; red dotted lines highlight the old batch of ovule primordia (C,D). (E-H) Distribution of *ProPIN1::PIN1-GFP* in placentae at floral stage 9a (E,F) and stage 9b (G,H). (F,H) Images merged with calcofluor white (cyan) stained cell wall images. The dotted rectangles in G,H indicate one small ovule with low PIN1-GFP signal levels between two large ovules with high PIN1-GFP signal. (I-N) Distribution of *R2D2* in placentae at floral stage 9a (I-K) and stage 9b (L-N). (I,L) The distribution of *DII-n3×Venus*; (J,M) the distribution of *mDII-ntdTomato*; (K,N) images merged with calcofluor white (blue) stained cell wall images. (E-N) White arrowheads indicate the young batch of ovule primordia; red arrowheads indicate the old batch of ovule primordia. Scale bars: 20 μm.

To confirm the asynchronous initiation of ovule primordia, we observed the expression of *ProCUC3::CFP* (Gonçalves et al., 2015) to show the boundaries between ovules and to indicate the number of ovule primordia forming at stage 9. When the ovule primordia forms at stage 9a, *ProCUC3::CFP* is expressed in the boundaries between each two ovule primordia; there are only a few boundaries formed, demonstrating that only four to seven ovules form at stage 9a (Fig. 2A,B). When more ovule primordia are formed at stage 9b, the clusters of *ProCUC3::CFP* signals are not evenly arranged, demonstrating that ovules are in different sizes (Fig. 2C,D).

It has been reported that PIN1 is expressed in ovule primordia (Benková et al., 2003; Galbiati et al., 2013). We observed the *ProPIN1::PIN1-GFP* signal during the ovule initiation process. At stage 9a, the PIN1-GFP signal clustered to the ovule protrusions and there were only a few signal clusters detected in the placenta (Fig. 2E,F). At stage 9b, there were different ranges and intensities of PIN1-GFP expression in different cell clusters (Fig. 2G,H), demonstrating that there are ovules of different sizes in the same placenta.

R2D2 combines with DII-n3×Venus and mDII-ntdTomato to show auxin gradients in which the absence of DII fluorescence marks auxin accumulation (Brunoud et al., 2012; Liao et al., 2015). We also observed the *R2D2* expression pattern. At stage 9a, there are four to seven regions on the placenta where no signal is detected, these regions represent the position of the ovule primordia (Fig. 2I-K). At stage 9b, there are more regions with no signal, and the signal intensity is not even, which means that more ovule primordia initiate and the ovules are not the same size (Fig. 2L-N).

Based on the *CUC3*, *PIN1* and *R2D2* expression regions, we demonstrate that there are only a few ovule primordia initiating at stage 9a and that other ovules initiate later. These data indicate that the ovule initiation is asynchronous in the same placenta.

### Ovule primordia develop asynchronously at subsequent stages

**Fig. 3.**
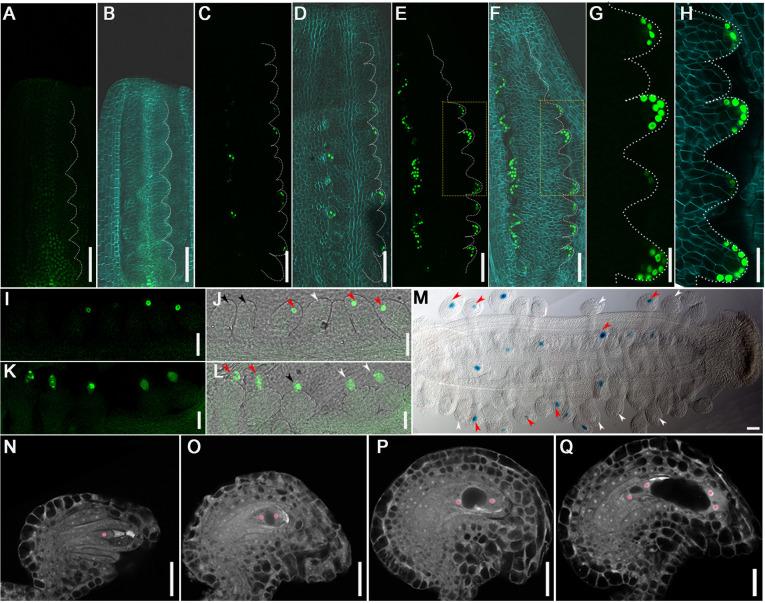
**Asynchronous ovule development during all stages in the same placentae.** (A-H) Expression pattern of *ProWUS::3×VENUS-N7* in placentae at stage 9b (A,B) and stage 9c (C-F). (G,H) Magnified views of the area outlined by the dotted rectangles in E,F. (A-H) Dotted line highlights ovule primordia. (B,D,F,H) Images merged with bright-field and cell wall staining by calcofluor white (cyan) images. (I-L) *ProKNU::KNU-VENUS* expression pattern shows ovules at MMC differentiation stage (I,J) and meiosis stage (K,L). Black, white and red arrowheads, respectively, indicate ovules with no signal, weak signal and strong signal in J, and ovules at MMC stage, meiophase and tetrad stage in L. (M) *ProFM1::GUS* expression pattern shows ovules at the FM identity stage. Red and white arrows indicate ovules with and without GUS signal, respectively. (N-Q) CLSM observation of different ovules in the same pistil at stage 12. Ovules are mainly at the two developmental stages of FG1 (N) and FG2 (O), or FG2 (O) and FG3 (P). There are a few ovules in FG4 (Q) stages. Pink highlights nuclei. Scale bars: 50 μm in A-F,M; 20 μm in G-L,N-Q.

We examined the expression pattern of different markers to indicate the stage of ovules in the same placenta after ovule primorida initiation. *WUSCHEL* (*WUS*) mRNA is expressed in the ovule at stage 9-10 (Groß-Hardt et al., 2002). We used the *ProWUS::3xVENUS-N7* transgene to observe the ovule primordia formation (Zhang et al., 2017). *WUS* was not expressed before stage 9b (Fig. 3A,B), but was expressed from stage 9c, and its expression level increased with ovule primordia elongation (Fig. 3C-F). The intensity and distribution of *WUS* mRNA differs among ovules in the same placenta (Fig. 3G,H), suggesting that asynchronous development of ovules continues at stage 9c-10.

KNUCKLES (KNU) is expressed in the MMC throughout meiosis (Payne et al., 2004; Zhao et al., 2018). At stage 10, the stage of MMC specification, the *ProKNU::KNU-VENUS* signal exhibit three patterns: no signal, weak signal and strong signal (Fig. 3I,J). All three levels of KNU-VENUS signal were observed in the same placenta (Fig. S2A). At stage 11, we also observed different patterns of KNU localization: one nucleus in MMCs and two to four nuclei in megaspore cells during meiosis in the same placenta (Fig. 3K,L; Fig. S2B). These results indicate that ovule development remains asynchronous at the MMC differentiation and meiosis stages.

Next, we observed the expression pattern of *FM1*, a marker gene of the functional megaspore (FM) (Huanca-Mamani et al., 2005). We found that the *ProFM1::GUS* transgene was expressed in some ovules at stages of FM specification and division (stage 12a-12b) (Fig. 3M). There are ovules with or without *ProFM1::GUS* signal in the same placenta (Fig. S2C), indicating that ovule development is still asynchronous at the FM specification stage.

Finally, we observed embryo sac (ES) development at stage 12 in the same placenta using optical sections with confocal laser scanning microscopy (CLSM) (Fig. 3N-Q) as described previously (Christensen et al., 1997). The ESs in the same placenta are mainly at two continuous stages between the FG1 and FG4 (Fig. S2D). These results suggest that ovule development remains asynchronous at the ES development stage, which is consistent with previous publications (Christensen et al., 1997).

In conclusion, ovule development is asynchronous from ovule primordia initiation to ES maturation, and ovules present in the same placenta are mostly at two continuous developmental stages. We speculate that ovule primordia initiation in different batches results in the asynchronous development of ovules in all subsequent stages.

### PIN1 polarity, auxin accumulation and auxin response correlate with ovule primordium initiation

**Fig. 4.**
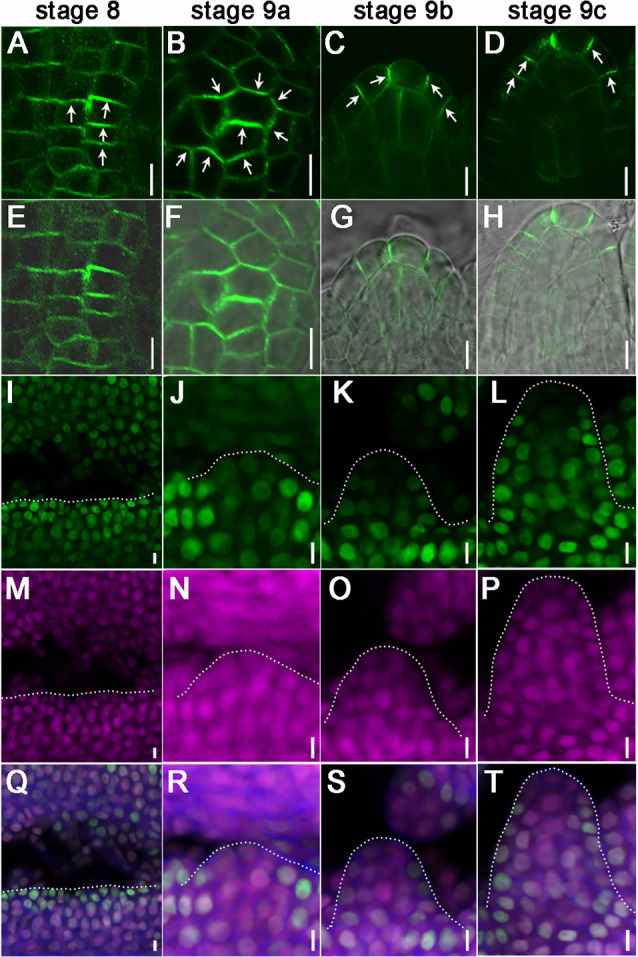
**Dynamic distribution of PIN1 and auxin in the process of ovule primordia initiation.** (A-H) *ProPIN1::PIN1-GFP* localization in one ovule primordium (A-D). (E-H) Images merged with the bright-field images. (A-D) Arrows indicate PIN1-GFP polar localization. (I-T) *R2D2* distribution in one ovule primordium. The distribution of *DII-n3×Venus* (I-L); the distribution of *mDII-ntdTomato* (M-P); images merged with calcofluor white (blue) stained cell wall images (Q-T). Dotted lines highlight the placenta or initiated ovule primordia. The top view of ovule primordia is shown in A,B,E,F. Scale bars: 5 μm.

Auxin plays an essential role in ovule primordia formation, so we examined auxin transport using *ProPIN1::PIN1-GFP*, auxin accumulation using R2D2 and auxin response using *DR5::NLS-eGFP* during ovule primordia initiation (Heisler et al., 2005; Brunoud et al., 2012; Liao et al., 2015). At stage 8, PIN1-GFP polarity is detected on the transverse sides of some placenta cells; these cells indicate the putative position of ovule primordia initiation (Fig. 4A,E; Fig. S3A). When the ovule primordia initiated at stage 9a-9b, PIN1-GFP was also found at the lateral sides of some placenta cells, the expression level was further increased and the signal was focused towards the primordia tips (Fig. 4B,C,F,G; Fig. S3B,C). In the existing ovules, PIN1-GFP signals were located predominantly on the sides of epidermis cell layers towards the ovule apex (Fig. 4D,H; Fig. S3D).

During ovule primordia initiation, auxin accumulation was detected by R2D2, and auxin response was detected using *DR5::NLS-eGFP* (Liao et al., 2015). At stage 8, the DII signal was expressed evenly in placenta cells and no DR5 signal was detected, indicating that no significant peak of auxin accumulation or response formed in the placenta, despite PIN1 expression (Fig. 4I,M,Q; Fig. S3E,I). At stage 9, the DII signal decreases, and DR5 signals appear in tip cells of the protrusions, indicating that the peak of auxin accumulation and response occurred in these cells after the ovule primordia initiation (Fig. 4J-L,N-P,R-T; Fig. S3F-H,J-L).

### NPA treatment inhibits ovule primordia initiation

The dynamic polarity of PIN1 is connected with the establishment of auxin distribution and maximum peak at primordia tips (Benková et al., 2003; Heisler et al., 2005; Galbiati et al., 2013; Cucinotta et al., 2020). But the dynamic of PIN1 polarity and auxin distribution of the ovule primordia have not been described in detail. To analyze how PIN1 polarity dynamics and auxin distribution connect with ovule primordia initiation, we applied an inhibitor of auxin polar transport, N-1-naphthylphthalamic acid (NPA), to inflorescences at stage 8-9. The 100 μM NPA treatment at the smallest pistils can affect gynoecium development, thereby decreasing ovule number (Nemhauser et al., 2000; Larsson et al., 2014). We treated the inflorescence apex with different concentrations of NPA and found that ovule primordia initiation was more sensitive than gynoecium development to NPA (Fig. S4).

**Fig. 5.**
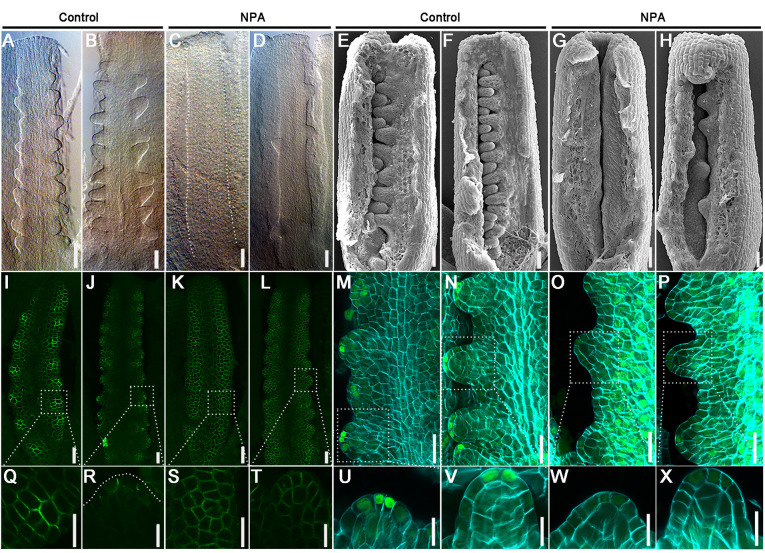
**NPA treatment inhibits the initiation of new ovule primordia via its effects on PIN1 localization and auxin response.** (A-H) Phenotypical analysis of ovule primordia initiation under NPA treatment for 24 h. Morphological observation of placentae by DIC (A-D) and SEM (E-H) at 2 DAT (day after treatment). Dotted lines indicate the placentae. (I-L) *ProPIN1::PIN1-GFP* expression and localization after treatment with the mock solution (I,J) and NPA (K,L) for 24 h. (M-P) *DR5::NLS-eGFP* expression and distribution (green) after treatment with the mock solution (M,N) and NPA (O,P) for 24 h, cell wall stained with calcofluor white (cyan). (Q-X) Magnified views of ovule primordia marked with dotted rectangles in I-P, respectively. (A,C,E,G,I,K,M,O,Q,S,U,W) Pistils treated at stage 8. (B,D,F,H,J,L,N,P,R,T,V,X) Pistils treated at stage 9. Similar results were obtained in three independent experiments. Scale bars: 20 μm in A-P; 10 μm in Q-X.

Under mock control treatment, ovule primordia initiate normally (Fig. 5A,B,E,F; Fig. S4A,B): PIN1 levels increase in placentae, PIN1 polarity points towards the primordia tip (Fig. 5I,J,Q,R) and DR5 response maxima form at the primordia tips during the initiation (Fig. 5M,N,U,V). In the NPA-treated samples, although the gynoecium development is normal, the initiation of new ovule primordia stops, the existing ovule primordia keep growing at low NPA concentrations but the ovule growth finally arrests in high concentrations of NPA (Fig. 5C,D,G,H; Fig. S4C-H). PIN1 expression dramatically decreases and PIN1 polarity disappears at stage 9a-9b under NPA treatment (Fig. 5K,L,S,T). An auxin response maximum still forms in primordia tips but the intensity is decreased (Fig. 5O,P,W,X). These findings highlight that normal PIN1 polar localization and auxin response are essential for young ovule primordia initiation, and also demonstrate that ovule primordia are initiated in different groups.

### Computational models predict asynchronous ovule primordia initiation

**Fig. 6.**
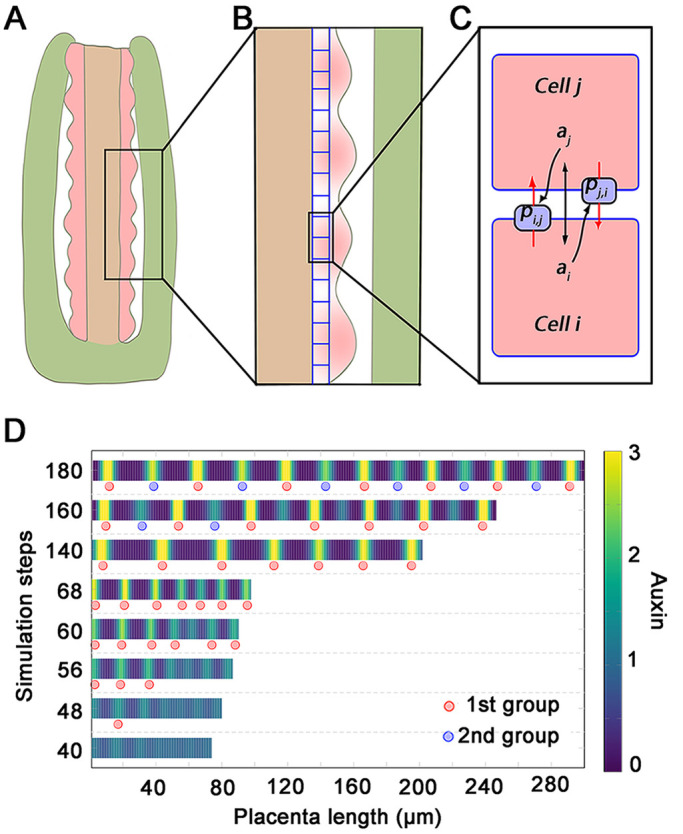
**Schematic diagram of the model and its principle.** (A) Pistil typically consists of the ovary wall (green area), placenta (pink area) and transmitting tract (brown area) at stage 9. (B) The distribution of auxin on the placenta is one-dimensional, i.e. only one layer of cells along the placenta direction is considered. (C) Auxin is transported between neighboring cells by PIN1, while PIN1 is polarized depending on auxin concentration in neighboring cells. (D) Placenta length and auxin distribution at different simulated time steps. The red dot indicates the potential initial position of the first group of ovules; the blue dot indicates the potential initial position of the second group of ovules.

We developed a computational model to predict ovule primordia initiation regulated by dynamic PIN1 localization and auxin distribution. The model simplified placenta elongation, auxin distribution and ovule primordia initiation into a one-dimensional line to simulate a perfect state (Fig. 6A,B). In the model, auxin was transported between neighboring cells by PIN1, while PIN1 polarization was determined by the auxin concentration in neighboring cells (Fig. 6C). Auxin was distributed almost evenly with tiny perturbations before initiation. Placenta cells expanded and divided to simulate placenta elongation (Fig. 6B). To smooth the spatial variation in auxin concentration, we performed a cubic spline interpolation on the simulation results. We showed the spatiotemporal distribution of auxin after interpolation and the length of the placenta (Fig. 6D). The simulation results showed that, in response to the action of PIN, the initial uniform distribution of auxin spontaneously changes to produce several localized maxima, which induce the auxin response and the initiation of the first group of ovule primordia (Fig. 6D; Movie 1). As the cells expand and divide, the placenta elongates (Fig. 6D; Movie 1). Subsequently, a second group of auxin maxima is formed, which induces the auxin response and the initiation of the second group of ovule primordia (Fig. 6D; Movie 1). Given the model assumptions, these results suggest that new ovule primordia initiation requires PIN1 polarity and the formation of localized auxin maxima, and that ovule primordia initiate in different groups tightly accompanied by the auxin response.

### BR promotes ovule primordia initiation

**Fig. 7.**
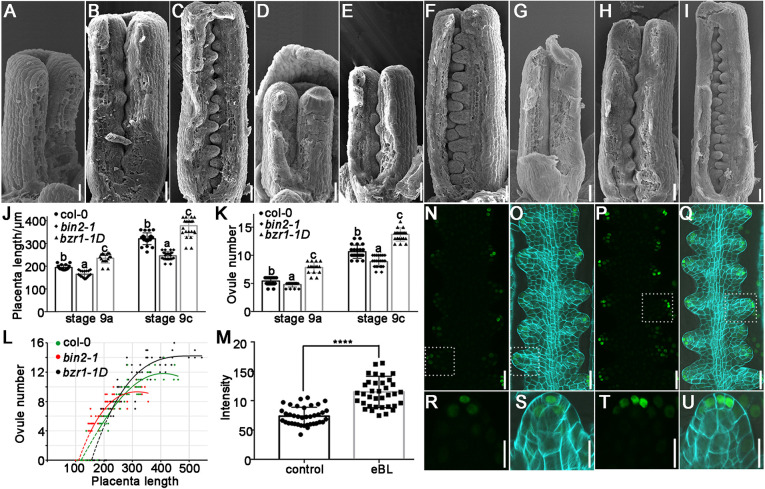
**Ovule primordia initiation and auxin response in BR-related mutant at stage 8-9.** (A-I) SEM images showing ovule primordia initiation in Col-0 at stage 8 (A), stage 9a (B) and stage 9c (C), in *bin2-1* at stage 8 (D), stage 9a (E) and stage 9c (F), and in *bzr1-1D* at stage 8 (G), stage 9a (H) and stage 9c (I). (J,K) Placenta length (J) and numbers of ovule primordia formed in each placenta (K) in wild-type and BR-related mutants at stage 9a and 9c. The data are mean±s.d., *n*>15 in every group; lowercase letters indicate statistically significant differences between different stages (*P*<0.05). (L) Analysis of the correlation between the number of ovule primordia and the length of the placenta at stages 8-10 in wild type and BR-related mutant. (M) Fluorescence intensity of *DR5::NLS-eGFP* after treatment with a mock solution and 2 μM eBL for 24 h. The data are mean±s.d., *n*>30 in every group (*t*-test; ****P<0.0001). (N-Q) *DR5::NLS-eGFP* expression and distribution (green) after treatment with the mock solution (N,O) and 2 μM eBL (P,Q) for 24 h; cell wall stained with calcofluor white (cyan) in P,Q. (R-U) Magnified views of ovule primordia marked with dotted rectangles in N-Q, respectively. Scale bars: 20 μm in A-I,N-Q; 10 μm in R-U.

Our previous research illustrated that BR influences ovule number through transcriptional regulation of the early ovule development-related genes *ANT*, *HLL* and *AP2* (Huang et al., 2013). To explore whether BR affects ovule initiation, we observed ovule primordia initiation in the BR-insensitive mutant *bin2-1* and the BR-enhanced mutant *bzr1-1D* (Li and Nam, 2002; Wang et al., 2002). At stage 8, no protrusions appear in the placentae of wild type, *bin2-1* and *bzr1-1D* (Fig. 7A,D,G). At stage 9a, the placental length is ∼180 μm in Col-0 when the first batch of ovule primordia initiate (Fig. 7B,J,L). At the same developmental stage, *bin2-1* and *bzr1-1D* have smaller and larger placentae, respectively (Fig. 7E,H,J,L). In addition, there is a lower and a higher number of ovule primordia in the first group in *bin2-1* and *bzr1-1D*, respectively (Fig. 7B,E,H,K). These results suggest that BR contributes to placenta elongation and the protrusion of the ovules of the first batch. Similar results were observed at stage 9c (Fig. 7C,F,I-K). The phenotype of the crowded ovule in *bzr1-1D* at stage 9 indicates higher ovule density (the ratio of the ovule number to the placenta length) (Fig. S5) (Jiang et al., 2020), and suggests that BR increases seed number not only through promoting placentae elongation but also through promoting ovule primordia initiation. Flowers treated with 2,4-epibrassinolide (eBL) at stage 8-9 exhibited increased DR5 signal, indicating a significantly enhanced auxin response (Fig. 7M-U). This result demonstrates that BR also affects the auxin response to promote ovule primordia initiation.

### Transcriptomic analysis of gene expression during ovule development

To obtain the global gene expression trends during ovule development, we collect samples of the gynoecium in developmental stages 9-10, 11 and 12 to perform a microarray assay. 4694 genes are identified as differentially expressed genes (DEGs) from 19,665 genes (fold change≥2.0 and *P*≤0.05) (Figs S6A and S7A,B; Table S1). Hierarchical clustering of the DEGs reveal there are relatively similar transcription patterns at stage 9-10 and stage 11, but different expression patterns are found at stage 12 compared with those at stages 9-11 (Fig. S6B). In addition, the enriched Kyoto Encyclopedia of Genes and Genomes (KEGG) pathways and Gene Ontology (GO) analysis indicate that vigorous hormone signaling and biosynthetic/metabolic processes are required during ovule development (Fig. S7C,D).

To explore the possible functions of the DEGs in ovule development, clustering analysis by STEM is used to further divide the DEGs into 12 clusters of clear and distinct expression profiles. The top six clusters are exhibited in Fig. S6C. Clusters 2, 3 and 6 contain genes highly expressed at stage 9-10, suggesting that these genes function during ovule initiation and development. Genes reported to influence these processes, such as *CUC*, *ANT*, *KNU* and *WUS*, are identified in DEGs, and their transcription patterns are consistent with the expression pattern of our marker lines or previous reports (Figs 2 and 3; Table S1) (Schneitz et al., 1998; Groß-Hardt et al., 2002; Payne et al., 2004; Gonçalves et al., 2015).

Our results also illustrated some regulators of shoot apical meristem (SAM) development and maintenance, such as *SHOOT MERISTEMLESS* (*STM*), *ASYMETRIC LEAVES 2* (*AS2*), *KNOTTED-LIKE FROM ARABIDOPSIS THALIANA2* (*KNAT2*) (Barton and Poethig, 1993; Lincoln et al., 1994; Semiarti et al., 2001). We also identified *WOX1*, *WOX12* and *WOX13* at stage 9-10, suggesting that these WOX genes play essential roles in ovule primordia formation (Table S1). The WOX gene family has been reported to regulate embryogenesis, stem cell homeostasis and organ formation (Laux et al., 1996; Haecker et al., 2004; Deveaux et al., 2008), suggesting that they are reasonable players in ovule initiation and development. Clusters 1, 4 and 5 contained genes highly expressed at stage 12, indicating that they play an important role in embryo sac development and ovule micropyle formation. For example, unfertilized embryo sac mutant (UNE), which exhibits defects in fertilization (Pagnussat, 2005), was identified in cluster 4 (Table S1). In addition to already identified genes, our DEGs also included homologs of known genes and completely new genes. Rapid alkalinization factor (RALF) family members, e.g. small peptide RALF4/19/34, play an important role during gametogenesis and fertilization (Haruta et al., 2014; Ge et al., 2017). We found that many other RALF family members were highly expressed at stage 12, suggesting these RALF peptides also participate in male-female interaction. Receptor-like kinases (RLKs), including POLLEN-SPECIFIC RECEPTOR-LIKE KINASE, ANXUR, MALE DISCOVERER and others, play essential roles in male-female communication (Takeuchi and Higashiyama, 2016; Wang et al., 2016; Ge et al., 2017). Other RLKs are highly expressed at stage 12, such as RLP31, MRH1 and FLS2, suggesting that these RLKs also function in male-female communication. Some DEGs have not been reported previously (Table S1) and other DEGs are currently uncharacterized (e.g. *MYB*, *WRKY*, *CYP*, etc.) but with limited clues, indicating new candidates and regulatory mechanisms for ovule development that are worthy of future investigation.

Plant hormones have been reported to play roles in gynoecium development, especially auxin, BR, CK and GA (Colombo et al., 2008; Reyes-Olalde et al., 2013; Zúñiga-Mayo et al., 2019; Cucinotta et al., 2020). Among the DEGs, we identified lots of hormone-related genes, including important components of their biosynthesis, transport and response, indicating the hormonal regulation in ovule development (Table S2). Hierarchical clustering of 39 auxin-related genes shows that most of the genes encoding AUX/IAA proteins were upregulated, and auxin response factors (ARFs) and auxin metabolic genes were downregulated (Fig. S6D; Table S2). The expression patterns of auxin biosynthesis genes and auxin polar transporters were different from stage 9 to stage 12, which implied that these genes functioned at different stages and took part in different events. For example, PIN1 was expressed at stage 9-10, which mainly coincides with ovule initiation, consistent with the *ProPIN1::PIN1-GFP* expression pattern (Fig. 2E–H; Fig. S6D; Table S2). Among the 21 BR-related genes, most BR synthesis genes were gradually downregulated, while BR response genes were mostly upregulated (Fig. S6E; Table S2), indicating that BR signaling remains active during ovule development and that the BR biosynthesis is feedback inhibited. Hierarchical clustering of 23 CK-related genes showed that the expression patterns of CK synthase, glucosyltransferase, CK receptors and CK response genes were gradually downregulated, and CK hydrolase and transport enzyme gene were upregulated, indicating that CK played essential roles in early ovule development and would be downregulated in late ovule development (Fig. S6F; Table S2). For GA-related genes, GA biosynthetic and response genes were highly expressed at stage 12-13, but genes in the GA signaling pathway were highly expressed at stage 9-10 (Fig. S6G; Table S2). Above all, hormone signaling is active throughout ovule development; each hormone is accurately modulated to regulate ovule development.

## DISCUSSION

### Asynchronous ovule primordia initiation is important for plant reproductive development

**Fig. 8.**
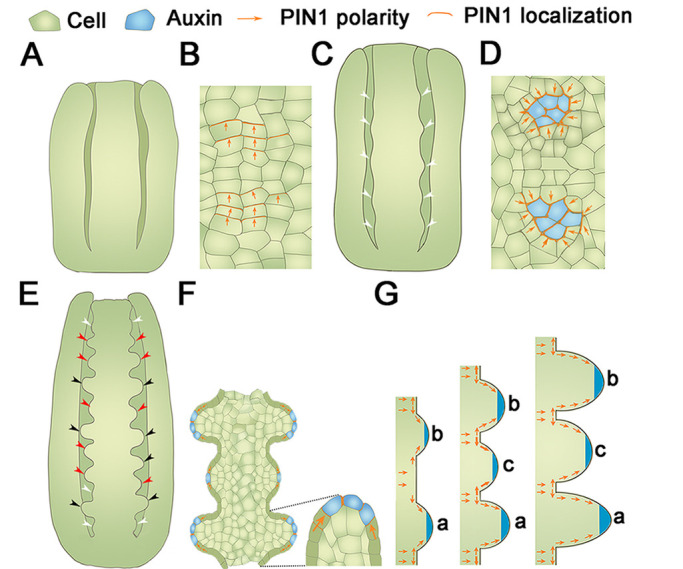
**Model of the ovule primordia initiation process, and expression patterns of PIN1 and auxin.** (A-F) The ovule primordia initiation process at stage 8 (A), stage 9a (C) and stage 9c (E); PIN1 polar localization and auxin maxima in ovule primordia at stage 8 (B), stage 9a (D) and stage 9c (F). White arrowheads indicate the O1; red arrowheads indicate the O2; black arrowheads indicate the O3. (G) The distribution and flow of auxin between ovule primordia throughout the placenta. Orange arrows indicate the PIN1 polarity.

Previous studies have mentioned ovule primordia formation (Bartrina et al., 2011; Huang et al., 2013; Gonçalves et al., 2015) and ovule development, such as the nucellus identity, integument initiation and embryo sac development (Villanueva et al., 1999; Erbasol Serbes et al., 2019). Here, we have observed the detailed process of ovule primordia initiation by DIC and SEM (Fig. 1). Our results show that ovule primordia initiate asynchronously in the same placenta, and that new ovule primordia initiate mainly between older neighboring ovule primordia (Fig. 8). The first batch of ovule primordia appears at stage 9a (Fig. 8C). Later, two groups of ovule primordia exist on the same placenta, and can easily be distinguished according to their size and shape (Fig. 8E). Statistical analysis demonstrated that ovules on the same placenta are mainly at two continuous developmental stages. The third batch of ovule primordia initiated normally at the bottom and top of the placenta, and differed significantly from the size and shape of the first two groups. Our hypothesis illustrates that the ovule population initiation is an interrelated process. Until now, published models mainly described single ovule primordium initiation and the signals between the primordium and its two boundaries (Galbiati et al., 2013; Cucinotta et al., 2020). Our hypothesis describes the relationship of neighboring ovule primordia during the initiation.

The close relationship between larger placental size and enhanced seed number in some mutants (*ckx3 ckx5* and *bzr1-1D*) indicates that placenta size is one factor that affects ovule primordia initiation (Bartrina et al., 2011). However, the limited examples cannot clarify whether a larger placenta causes more primordia initiation in the first and the second rounds, or whether there are more rounds of primordia initiation. Here, we demonstrate that the increased ovule number in the BR-signal-enhanced mutant comes from larger placental size and increased ovule primordia initiation in the first and second batches (Fig. 7). Microscopic analyses show that the larger and smaller ovules are not arranged evenly (Fig. 1C,D,H,I). This could be reasonably explained by the insufficient boundary of new ovules between some larger ovules. If the boundary is large enough, two young ovule primordia will initiate.

### Ovule primordia continue to develop asynchronously at subsequent stages

Although previous studies indictaed that ovules at different stages (mainly 2-3) exist in the same gynoecium (Christensen et al., 1997), the reason remained unclear. Our results reveal there are different expression patterns of marker genes at different ovules in the same placenta, indicating that ovule primordia initiates mainly in two batches and grows out at a similar speed, leading to the ovules in the same gynoecium developing asynchronously from ovule primordia initiation (stage 9a) to embryo sac maturation (stage 12c) (Fig. 3). Previous reports have shown that pollen tubes preferentially guide to ovules at the middle pistil but not to the topper ovules (Feng et al., 2019). A reasonable explanation is that the pollen tube guiding sequence correlates with the ovule maturing sequence. The asynchronous initiation of ovule primordia is significant in plant reproductive development. The existing ovules can survive environmental stress but new ovules cannot grow out, which would be the effective way for the plant to allocate nutrition and control the number of offspring. Previous reports did not completely exclude the possibility that ovules initiated simultaneously but grew out at different speeds. Our results indicate the high possibility of asynchronous ovule initiation because only 5-7 ovules protrude firstly in one placenta, and more ovules of different sizes and shapes exist in the same placenta and at the same time at floral stage 9b-9c (Figs 1 and 2), and so on.

Transcriptomics analysis illustrates that the expression of hormone-related genes is accurately regulated during this process, indicating that those hormones maintain the normal developmental process of ovules (Fig. S6). Auxin is an important player in different processes of ovule development, indicating auxin functions in the whole process. BR signal is also activated during whole-ovule development, and BR promotes ovule initiation by enhancing placenta elongation and the auxin response (Fig. 7), as well as by transcriptional regulation of genes involved in early ovule development (Huang et al., 2013). CK promotes placenta activity to establish supernumerary ovules at early stages (Bartrina et al., 2011). GA inhibits ovule primoridia initiation. Transcriptomics analyses of the placenta were carried out in the previous studies (Skinner and Gasser, 2009; Matias-Hernandez et al., 2010); our systemic analysis of the whole developmental processes of ovules identified new candidate genes involved in ovule development. The DEGs identified by our transcriptomics analysis largely overlap with the DEGs listed by Skinner and Gasser (2009), indicating that our transcriptional analysis worked well. Compared with the genes expressed in placentae (identified by Matias-Hernandez et al., 2010), we identified 5086 new genes involved in ovule initiation in stage 9-10 (S1). Some of them are involved in pattern formation, such as ASYMMETRIC LEAVES2-LIKE1, FASCIATA1 (FAS1), FAS2, ULTRAPETALA2 and POLTERGEIST-LIKE1 (Kaya et al., 2001; Chalfun-Junior et al., 2005; Song et al., 2006; Monfared et al., 2013), indicating that they are involved in ovule primordia initiation and formation. In addition, the DEGs in ovule primordia initiation and in magasporogenesis/megagametogenesis are very different. The genes highly expressed in ovule primordia initiation have similar characteristics to meristem tissue, which is repressed in ovule development, suggesting that the genes regulating ovule initiation might be different from the genes regulating ovule development.

### Homeostasis of PIN1 and auxin flow correlate asynchronous ovule primordia initiation

Unlike the process of lateral root primordia initiation from pericycle cells, ovule primordia initiate from the subepidermal layers by periclinal division (Vaughan, 1995). Previous research has reported that new primordium initiates between the old primordia in SAM (Heisler et al., 2005; Smith et al., 2006). After initiation of older primordia, new primordium is identified in the enlarged area between old primordia (Heisler et al., 2005). PIN1 expression gradually increases in new primordium and its neighboring cells, and PIN1 polarity points to the new primordium (Heisler et al., 2005). DR5 signal gradually appears at the top of the primordium (Heisler et al., 2005). After protrusion of the primordium, the expression of PIN1 decreases significantly, and the polarity of PIN1-GFP in epidermal cells at the base of the primordium reverses towards the center of meristem and adjacent areas (Heisler et al., 2005). DR5 signal continues to be expressed during the initiation process (Heisler et al., 2005).

Whereas the SAM is dome shaped, the placenta is the linear structure and the ovule primordia are distributed linearly among the placenta, whereas leaf and flower primordia are distributed among a circle of the central zone of the SAM. Our results reveal the dynamic distribution of PIN1 in placenta. During ovule primordia initiation, PIN1 expression increases on the transverse and lateral sides in some placental cells, indicative of auxin flow to form localized maxima, thus triggering the initiation of the first group of ovule primordia (Fig. 8A,B,G). After the protrusions, PIN1 polar distribution travels towards to primordia tips; thus, auxin accumulates there (Fig. 8C,D,G). As the placenta grows, PIN1 polarity may be reversed in old primordia and new PIN1 polarity occurs in the placental cells between older primordia (i.e. boundaries), which consequently initiates new protrusions (Fig. 8E-G). The computational model mimics these processes (Fig. 6). New primordium is initiated within the elongated boundary between neighboring older primordia in the SAM and placenta, indicating that there is a conserved mechanism in ovule primordia initiation. When gynoecium is treated with NPA, PIN1 polar distribution is dramatically reduced and auxin response maxima still form in existing primordia tips but at a much lower level (Fig. 5). Importantly, there is no re-localization of PIN1 to the boundaries, which arrests new ovule primordia initiation, although the old primordia can keep growing (Fig. 5; Fig. S4).

Our results also illustrate that modified BR signals change the placenta size and ovule initiation. The decreased ovule density of *bin2-1* and the increased ovule density of *bzr1-1D* (Fig. 7J,L) indicate that BR not only promotes placenta elongation, but also stimulates ovule primordia initiation. Our previous work reported that BR positively regulated ovule number through transcriptional regulation of early ovule development-related genes *ANT*, *HLL* and *AP2* (Schneitz et al., 1998; Huang et al., 2013). Here, we have found another way for BR to promote ovule initiation: by strengthening auxin response. The interaction of BR with auxin in root development has been well investigated (Hardtke, 2007; Cho et al., 2014; Oh et al., 2014; Tian et al., 2018; Sun et al., 2020), and our results reveal that the integration of BR and auxin promotes ovule development, indicating that the interaction of BR and auxin contributes to another developmental process.

In conclusion, our results show that ovule primordia initiate asynchronously. The accurate localization of PIN1, the formation of local auxin response maxima and elongation of the placenta are the main factors that determine this asynchrony initiation. Hormone signaling remains active and is modulated accurately to regulate ovule development.

## MATERIALS AND METHODS

### Plant material and growth conditions

*Arabidopsis thaliana* (Columbia-0) from ABRC (http://www.arabidopsis.org/) was used as wild-type or transgenic material, and plants used in this study include *bzr1-1D* (Wang et al., 2002), *bin2-1* (Li and Nam, 2002), *ProPIN1::PIN1-GFP* (Heisler et al., 2005), *ProWUS::3xVENUS-N7* (Zhang et al., 2017) and *ProKNU::KNU-VENUS* (Zhao et al., 2018), which have been described previously. Plants were grown in a 10:10:1 mix of peat substrate: vermiculite:perlite under a 16 h-light/8 h-dark photoperiod at 22±2°C. For growth on plates, surface-sterilized *Arabidopsis* seeds were placed on half-strength Murashige and Skoog (MS) basal medium. Plates were kept at 4°C for 3 days, then transferred to a growth chamber (Percival) with a 16 h-light/8 h-dark photoperiod at 22°C. The wild-type plants were transformed by the floral dip method using *Agrobacterium tumefaciens* strain GV3101.

### Plasmid construction and plant transformation

The *ProFM1::GUS* construct was generated using one-step cloning technology (Vazyme). The 880 bp sequence upstream of At4g12250 (*FM1*) was used as its promoter (*ProFM1*), as described elsewhere (Huanca-Mamani et al., 2005). *ProFM1* was cloned from genomic DNA with the primers proF and proR (proF, CGACTCTAGA**GGATCC**CATACTAGCATGTATCCAC; proR, CCGTACCCGG**GGATCC**TCGGTGGAACTTTATCGGTTT; bold nucleotides indicate the recognition sites of restriction endonuclease) containing the BamHI restriction site. The fragment was subsequently cloned into *pBI101.3* (Jefferson et al., 1987).

### Ovule analysis

For the wild type and mutants, pistils were dissected from fresh flowers at stages 8-12 under a stereoscope microscope. For DIC observations, pistils were placed in a drop of chloral hydrate solution (chloral hydrate: H_2_O: glycerol, 8:3:1) until the pistils cleared. Cleared pistils were observed under a microscope (Zeiss Axio Imager M2) with DIC optics. For SEM observations, pistils were fixed in 4% (v/v) glutaraldehyde, washed with phosphate buffer, then dehydrated using an ethanol gradient. The dehydrated pistils were coated with gold-palladium before observation under a S3400II SEM (Hitachi). CLSM of ovules was performed as described elsewhere (Christensen et al., 1997; Shi and Yang, 2011).

For GUS staining, the whole inflorescence was collected and fixed in ice-cold 80% (v/v) acetone and then vacuum infiltrated for 10 min. Tissues were then washed with GUS staining solution without X-Gluc twice and then incubated in GUS staining solution with 2 mM X-Gluc overnight at 37°C. Tissues were subsequently decolored in 70% (v/v) ethanol and then observed and photographed under a Zeiss Axio Imager M2 microscope with DIC optics.

### Fluorescent and confocal microscopy

For confocal fluorescence microscopy, pistils from flowers at stages 8-12 were fixed in 4% PFA for 1 h, then cleared in Clearsee reagent for 1 week as described previously (Kurihara et al., 2015). For cell wall staining, the cleared pistils were stained with calcofluor white (Fluorescent brightener 28; Sigma-Aldrich) for 1 h, as described elsewhere (Ursache et al., 2018). Pistils in a drop of Clearsee reagent was gently pressed under a coverslip on a conventional slide and then observed under a TCS SP8 microscope (Leica). The excitation and emission wavelengths were as follows: calcofluor white, excitation at 405 nm and emission at 425-475 nm; CFP, excitation at 445 nm and emission at 455-505 nm; strong VENUS and GFP, excitation at 488 nm and emission at 505-550 nm; tdTomato, excitation at 561 nm and emission at 575–620 nm.

### NPA and eBL treatment

For NPA and eBL treatments, we sprayed inflorescences with 10 μM NPA, 100 μM NPA (Sigma-Aldrich) or 2 μM eBL (Sigma-Aldrich) containing 0.01% (v/v) SilwetL-77. The NPA was dissolved in dimethyl sulfoxide to a stock concentration of 100 mM; mock treatments were performed with distilled water containing 0.1% (v/v) dimethyl sulfoxide and 0.01% SilwetL-77. The eBL was dissolved in ethanol to a stock concentration of 10 mM; mock treatments were performed with distilled water containing 0.2% (v/v) ethanol and 0.01% SilwetL-77. After the old flowers were removed, the remaining inflorescence was sprayed twice (in the morning and at dusk) on the first day (Li et al., 2005; Nole-Wilson et al., 2010), then kept moist in a 50 μl centrifuge tube with the same chemical solution as described previously (Li et al., 2018). After 24 h of treatment, plants were sprayed with distilled water to wash away the chemicals. The flowers from *ProPIN::PIN-GFP* and *DR5::NLS-eGFP* plants at stage 9 were dissected immediately for confocal analysis. DIC and SEM analyses were assessed after 3 or 7 days after first spraying.

### Auxin transport model in the one-dimensional placenta

In our model, cells are arranged on a one-dimensional line. The auxin is uniformly distributed in a cell, and its concentration in cell * *is denoted by *a*_*i*_. The auxin efflux carrier PIN1 is unevenly distributed at the cell *i* membrane, and its density in the membrane of cell *i* towards neighboring cell *j* is denoted by *p*_*i*,*j*_. The change in auxin concentration in cell *i*(*a*_*i*_) is described elsewhere (Jönsson et al., 2006; Smith et al., 2006; Fujita and Kawaguchi, 2018).(1)
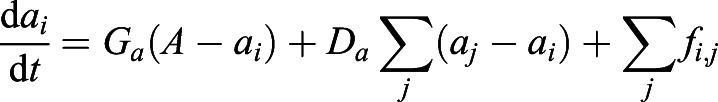
and(2)
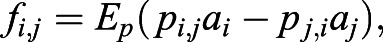
where cell *j* is the neighbor of cell *i*, *G*_*a*_ is the degradation rate, *A* is related to the synthesis rate, *D*_*a*_ is the diffusion coefficient, *E*_*p*_ is the efficiency of the PIN1 efflux carrier, and *f*_*i*,*j*_(=*f*_*j*,*i*_) is the net flow of auxin by PIN1 from cell *i* to cell *j*, consisting of auxin efflux and influx. The first term of the right-hand side of Eqn 1 indicates that auxin is constantly synthesized and degraded at a constant rate; the second term indicates that auxin is transported by PIN1; the third term represents the diffusion of auxin between neighboring cells.

The change in PIN1 density (*p*_*i*,*j*_) is described as follows:(3)
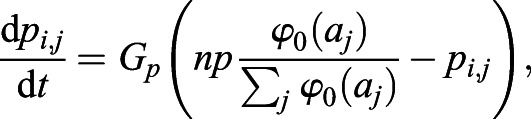
where *G*_*p*_ is the degradation rate, *n* is the number of neighboring cells, *p* is a constant related to PIN1 density and φ_0_(*a*_*j*_) is the regulatory function for PIN1 polarization (Fig. 5C). PIN1 is localized to the cell membrane, depending on the auxin concentration in neighboring cells and is degraded at a constant rate. The total amount of PIN1 in cell *i*, 
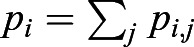
, satisfies the following equation:(4)
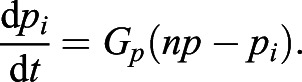


The above equation indicates that the stable equilibrium of *p*_*i*_ is *np*. Thus, equilibria of *a*_*i*_ and *p*_*i*,*j*_ are given, respectively, by the following:(5)
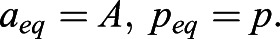


When *G*_*p*_ is sufficiently large, *p*_*i*,*j*_ quickly approaches equilibrium:(6)
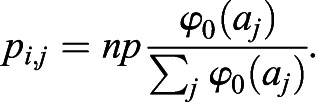
To simplify the model, φ_0_(*a*_*j*_)=*a*_*j*_ and Eqns 1-3 can be simplified as follows:(hbox7)
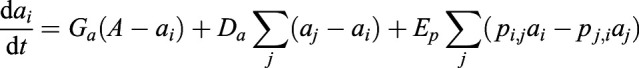
and(8)
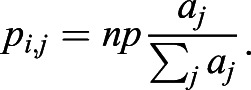
In this paper, auxin-regulated ovule development is simplified to a one-dimensional periodic boundary problem. Therefore, *j*=*i*±1 and *n*=2 in Eqns 7 and 8.

### Cell growth and division

The growth of the placenta is a macroscopic manifestation of cell growth and division. Therefore, to simulate placental growth, we need to consider cell growth and division. As our model is one-dimensional, we only consider the growth and division of cells along the length of the placenta. Cell growth satisfies the following equation:(9)
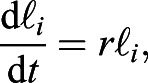
where ℓ_*i*_ is the length of the cell and *r* is the growth rate of cells, which is usually associated with auxin levels in the cells.

As the cells grow, the cell length, ℓ_*i*_, continues to grow. When ℓ_*i*_ is greater than a division threshold ℓ_div_, the cell divides into two equal-length daughter cells, both of which share the auxin of the mother cell:(10)
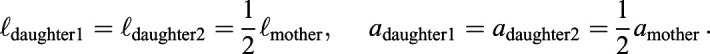


The model was implemented in MATLAB (https://www.mathworks.com). The differential Eqns 7 and 9 were solved by the difference method. At the initial time, the simulation system consisted of 50 cells of length 1, each with an auxin level of *a*=0.95+0.03(1−*i*/50)^2^+0.02*θ*, where *θ* is a random number uniformly distributed from 0 to 1. This will result in a slightly higher initial auxin level at the base than at the apex. We used the following parameters in the model: simulation time step Δ*t*=5×10^−4^, *D*_*a*_=0.5, PIN1 density constant *p*=1, efficiency of PIN1 efflux carrier *E*_*p*_=1, auxin degradation rate *G*_*a*_=0.1, cell length threshold ℓ_div_=4 and cell growth rate *r*=0.01.

### Microarray analysis

The microarray experiment samples were collected from wild type. More than 3 μg pistils was collected at stage 9-10 (S1), stage 11 (S2) and stage 12 (S3) with three biological replicates. The Agilent Arabidopsis (V4) Gene Expression Microarray (4*44K, Design ID:021169) was used in the experiment. Microarray experiments were conducted by OE Biotech using an Agilent Microarray Scanner G2505C. The original data were extracted from the scanned images using Feature Extraction software 10.7.1.1 (Agilent Technologies) and imported into Genespring software 13.1 (Agilent Technologies), using the quantile method to standardize the results. The results include the original signal values, normalized signal values and detailed annotation information. The DEGs were identified by the fold change of normalized signal values≥2 and *P*≤0.05 (*t*-test). The hierarchical clustering analysis, KEGG pathways and Gene Ontology enrichment of DEGs was produced by the OE Biotech Analysis Team.

### Quantification and statistical analysis

Placenta and ovule length measurements were made using ImageJ (https://imagej.nih.gov/ij/). In each related experiment, the number of repeats (*n*), sample sizes and *P*-values are indicated in the figure legends or in the Results section. Statistical significance was calculated using Student's *t*-test or one-way ANOVA with Prism7 (https://www.graphpad.com/scientific-software/prism/) and is indicated in the legends.

## Supplementary Material

Supplementary information
