# Supplementary material for: Asynchrony of ovule primordia initiation in Arabidopsis
Source: Development. 2020 Dec 23;147(24):dev196618. doi: 10.1242/dev.196618 (PMC7774900; doi:10.1242/dev.196618)
Supplement: Supplementary information [file develop-147-196618-s1.pdf]

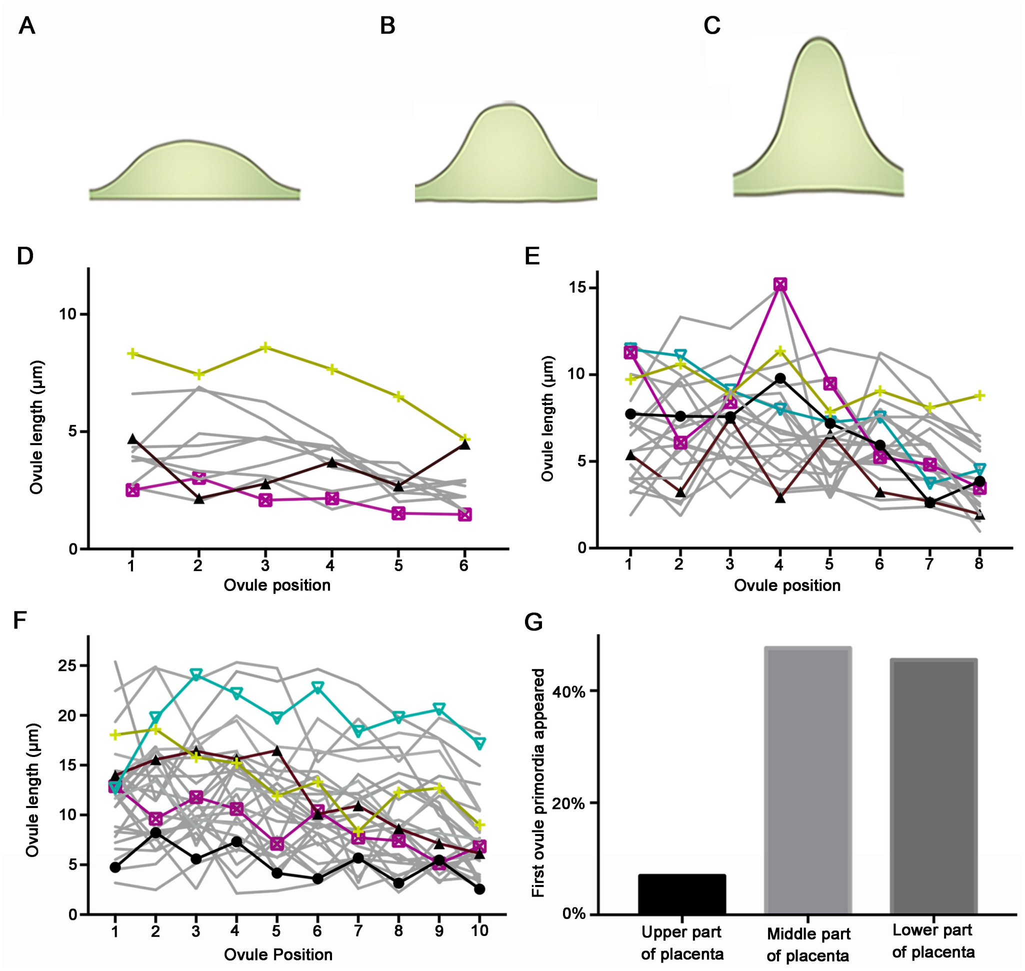

Figure S1. Quantification of ovule primordia height in different placentae at representative stages.

(A–C) The sketch of different ovule primordia shapes at stage 9 to stage 10: O1 shown small-bump-shaped (A), O2 shown dome-shaped (B), and O3 shown finger-shaped (C).

(D) Ovule primordia height at stage 9a: 6 ovules per placenta (*n*=11).

(E) Ovule primordia height at stage 9b: 8 ovules per placenta (*n*=24).

(F) Ovule primordia height at stage 9c: 10 ovules per placenta (*n*=32).

(G) Proportions of parts of the placenta where the first ovule protrudes (*n*=187).

Differently colored lines highlight the representative placenta in (D–E).

Development • Supplementary information

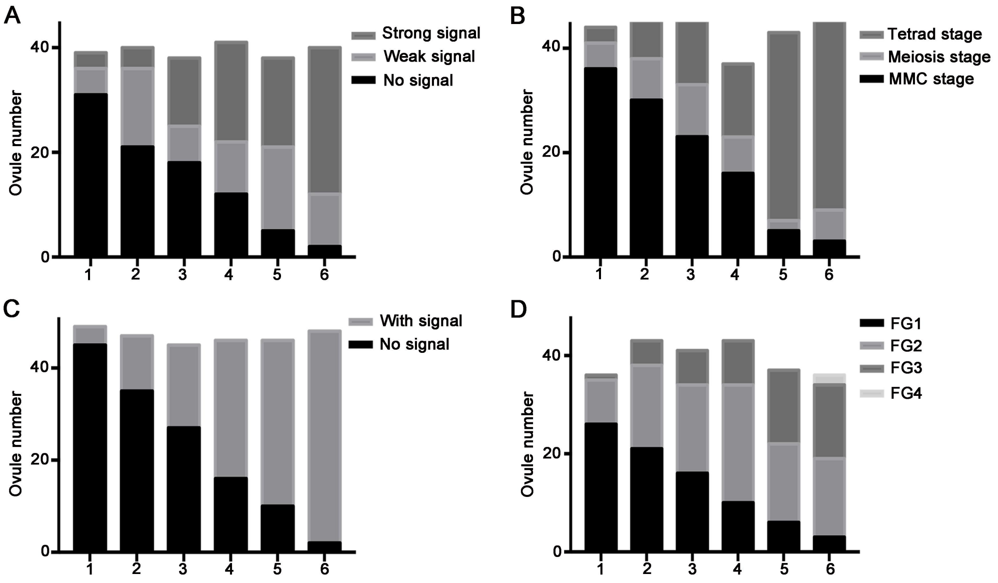

Figure S2. Quantification of ovule number with different signals at different stages.

(A) Representative pistils at megaspore mother cell (MMC) differentiation stage, according to KUN-VENUS expression pattern (*n*=15).

(B) Representative pistil at the meiosis stage, according to KUN-VENUS expression pattern (*n*=15).

(C) Representative pistil at function magaspore (FM) differentiation stage, according *ProFM1::GUS* expression pattern *(n*=46).

(D) Representative pistil at the meiosis stage, according to CLSM observations (*n*=10).

Every column represents an independent pistil in (A–D).

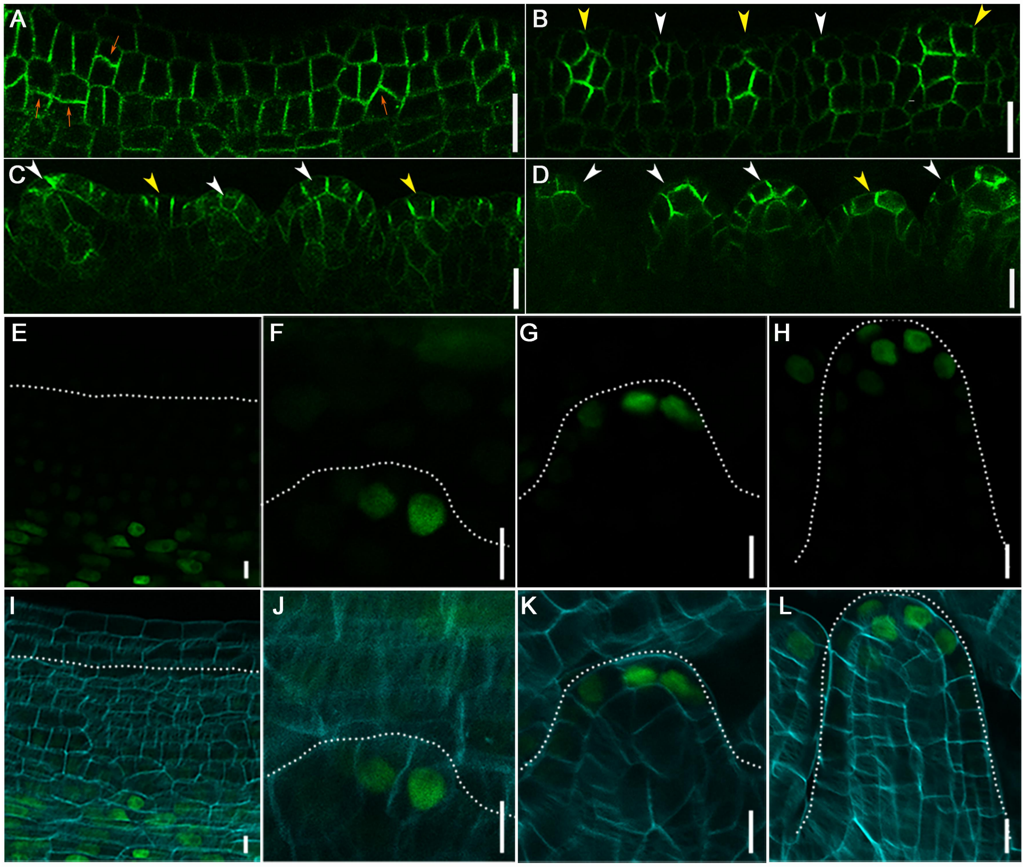

Figure S3. The distribution of *ProPIN::PIN-GFP* and *DR5::NLS-eGFP* in ovule primordia initiation process.

(A–D) *ProPIN1::PIN1-GFP* distribution in placentae at stage 8 (A), stage 9a (B), stage 9b (C), and stage 9c (D). Orange arrows point the cells in which the division direction changing, yellow arrowheads mark the young ovule primordia, white arrowheads mark the old ovule primordia (B–D).

(E–L) *DR5::NLS-eGFP* level and distribution. (E–H) *DR5::NLS-eGFP* merged with calcofluor white (cyan) stained cell wall. Dotted lines highlight the placenta in (E and I) and initiated ovule primordia in (F–H) and (J–L).

Bars = 20 µm in (A–D), 5 µm in (E–L).

Development • Supplementary information

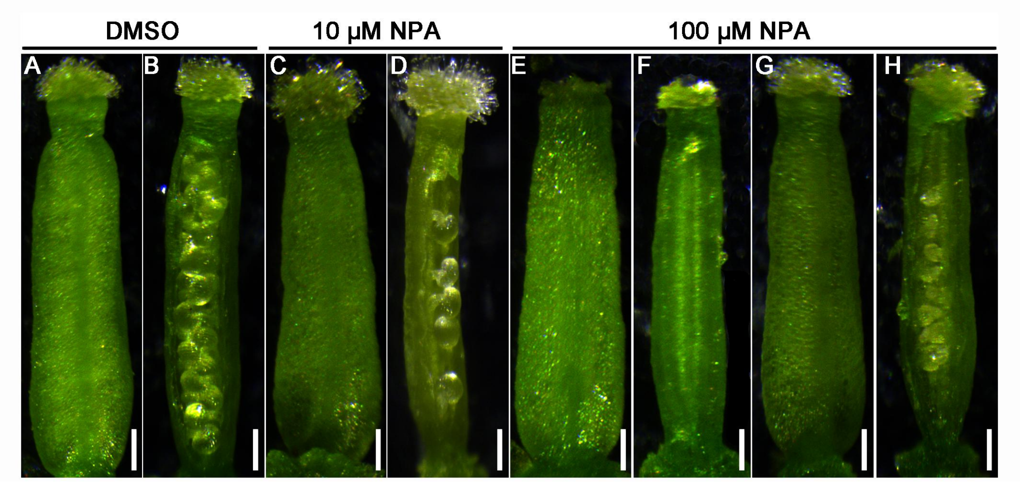

Figure S4. NPA treatment in different concentrations.

(A–B) The gynoecium (A) and ovules (B) at flower developmental stage 12 under DMSO treatment.

(C–D) The gynoecium (C) and ovule (D) at flower developmental stage 12 under 10 μM NPA treatment.

(E–H) The gynoecium (E, G), placenta (no ovule) (F), and ovule (H) at flower developmental stage 12 under 100 μM NPA treatment.

The gynoecium was harvested for observation at 7 days after treatment.

Bar = 100 μm.

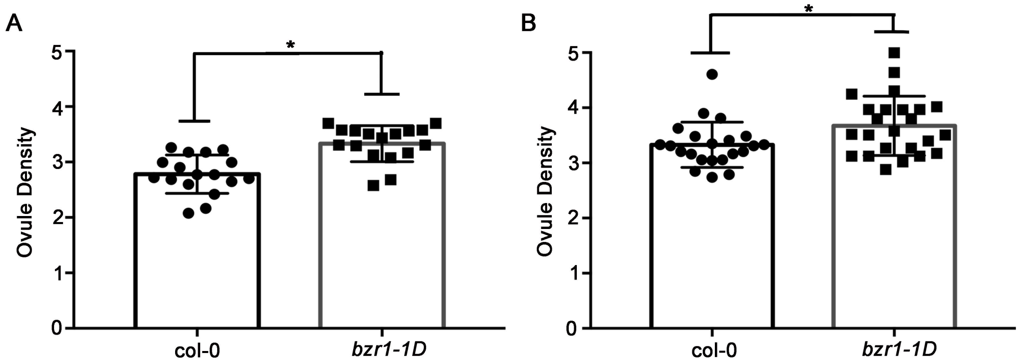

Figure S5. Ovule density in *bzr1-1D*.

(A) Ovule density at stage 9a, ovule density means the ratio of the ovule number to the placenta length per 100 μm.

(B) Ovule density at stage 9c.

The data are mean ± s.d.; n>15 in every group (one-way ANOVA; *P-value* < 0.05).

Development • Supplementary information

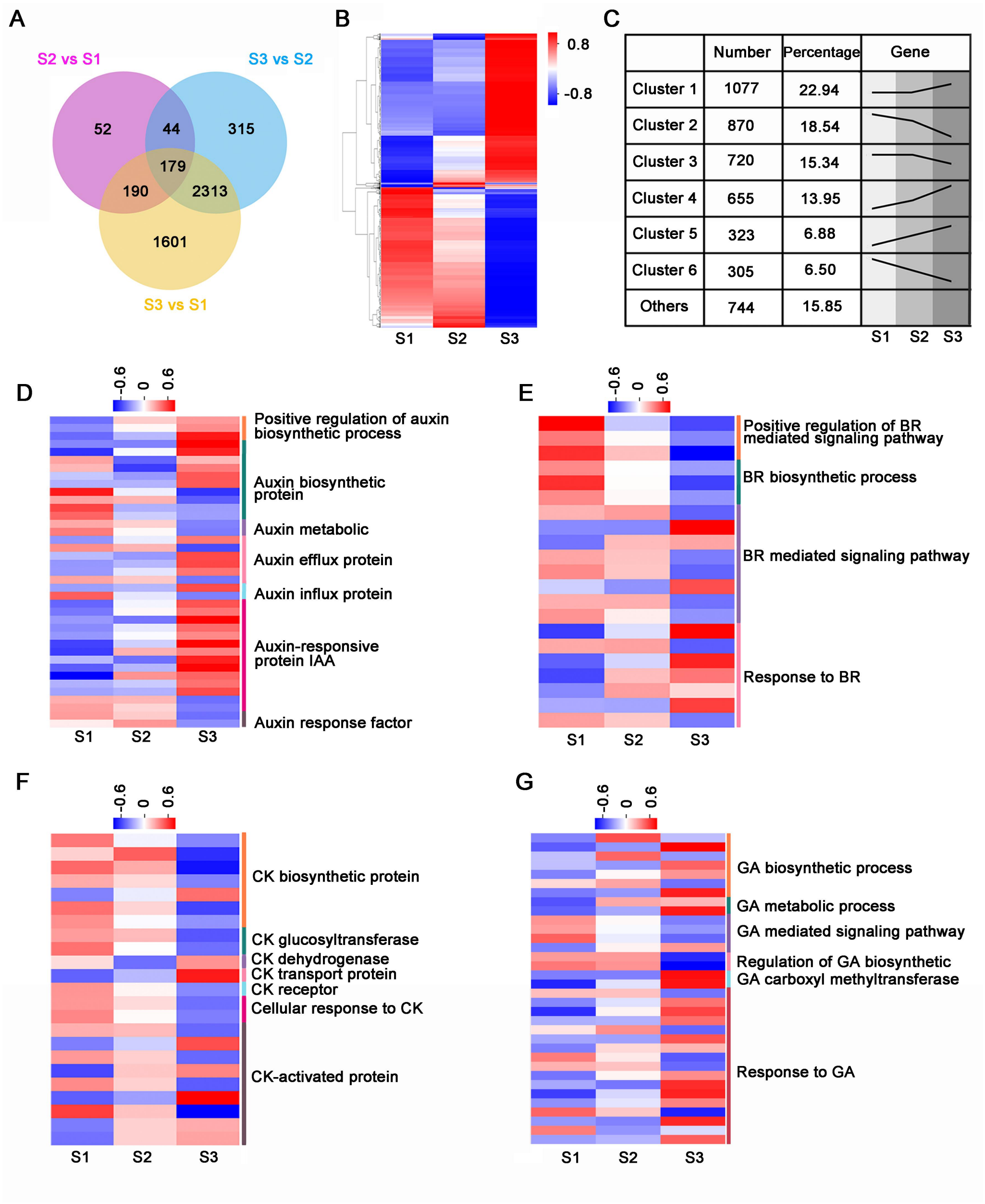

Figure S6. Microarray Analysis of the differentially expressed genes (DEGs) in ovule development at stage 9–10 (S1), stage 11 (S2), and stage 12 (S3).

(A) The number of DEGs between S1, S2, and S3.

(B) Heatmap of DEGs in S1, S2, and S3. The scale bar indicates the normalized signal value.

(C) These DEGs are clustered into main six clusters (1–6) based on their expression patterns in (B).

(D–G) Heat map visualizes the expression patterns of DEGs in the auxin signaling pathway (D), brassinosteroid signaling pathway (E), cytokinin signaling pathway (F), gibberellin signaling pathway (G). The scale bar indicates the normalized signal value.

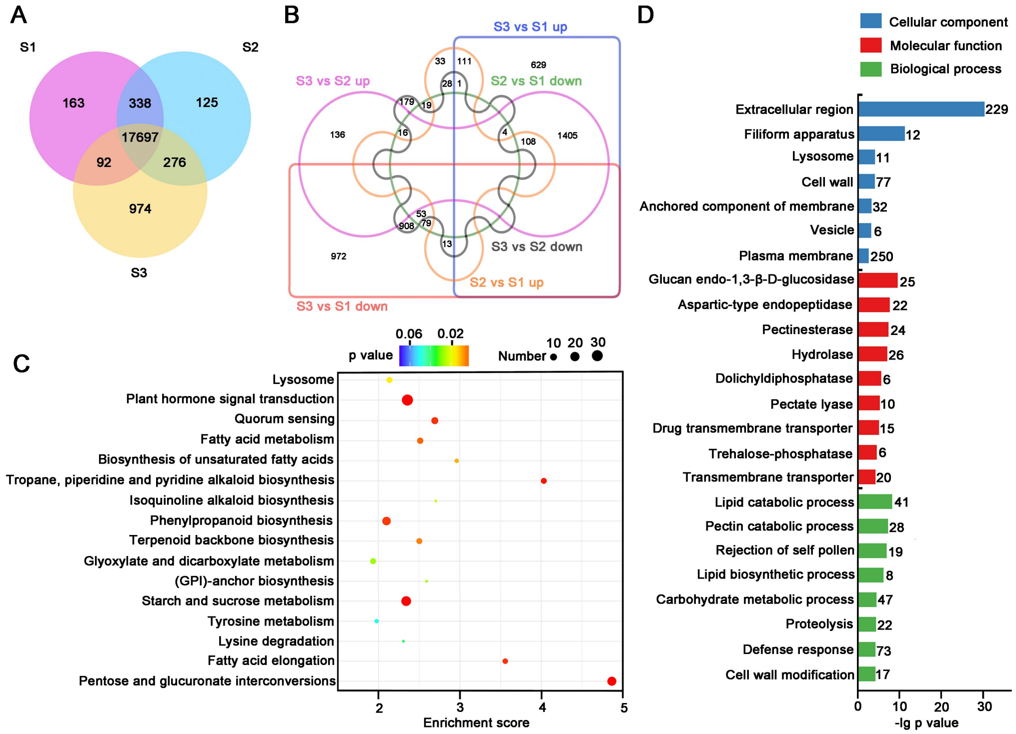

Figure S7. Overview of the detected genes and transcriptomic analysis of DEGs of pistils at stage 9–12.

(A) All genes we detected in stage 9–10 (S1), stage 11 (S2), stage 12 (S3).

(B) Overview of DEGs (upregulated and downregulated) between S1, S2, and S3.

(C) KEGG analysis shows that diverse pathways are enriched among the DEGs between S1, S2, and S3.

(D) Gene Ontology analysis of the DEGs between S1, S2, and S3. The numbers next to the column indicate the gene number.

Development • Supplementary information

Table S1. List of Differentially Expressed Genes in S1-S3.

Click here to Download Table S1

Table S2. Genes involved in auxin, brassinosteroid, cytokinin, and gibberellin signaling among the DEGs.

Click here to Download Table S2

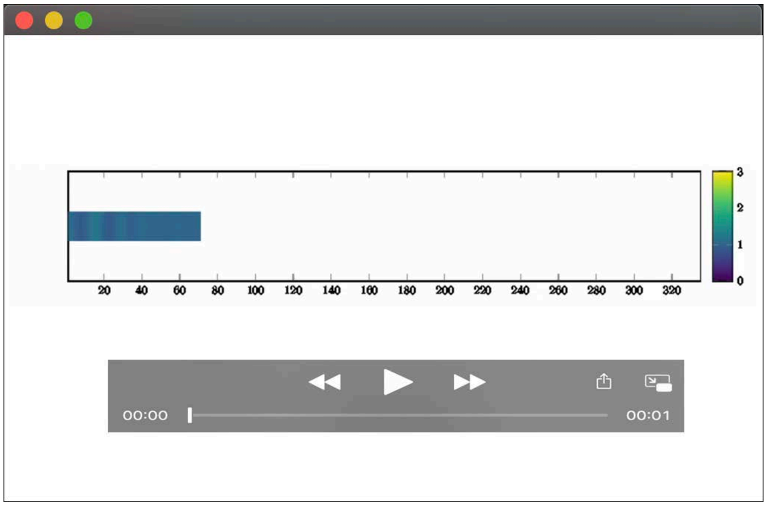

Movie 1. A computational model for auxin-regulated ovule initiation
